# Expert opinions on pharmacological symptomatic treatment of behavioral symptoms in frontotemporal dementia: A survey of the Neuropsychiatric International Consortium on Frontotemporal Dementia (NIC‐FTD)

**DOI:** 10.1111/ene.16537

**Published:** 2024-11-28

**Authors:** Dirk van Paassen, Luc Hartog, Sterre de Boer, Everard Vijverberg, Simon Ducharme, Yolande Pijnenburg, Alexander Santillo

**Affiliations:** ^1^ Alzheimer Center Amsterdam, Neurology Vrije Universiteit Amsterdam Amsterdam The Netherlands; ^2^ Amsterdam Neuroscience Neurodegeneration Amsterdam The Netherlands; ^3^ School of Psychology and Brain & Mind Centre University of Sydney Sydney New South Wales Australia; ^4^ Department of Psychiatry Douglas Mental Health University Institute, McGill University Montreal Quebec Canada; ^5^ McConnell Brain Imaging Centre, Montreal Neurological Institute McGill University Montreal Quebec Canada; ^6^ Department of Clinical Sciences, Clinical Memory Research Unit, Faculty of Medicine Lund University Lund Sweden

**Keywords:** behavioral symptoms, behavioral variant frontotemporal dementia, bvFTD, clinical practice, genetic, pharmacological treatment, sporadic, symptomatic treatment

## Abstract

**Background and purpose:**

Behavioral variant frontotemporal dementia (bvFTD) is essentially characterized by progressive changes in personality and cognition. Clinically, bvFTD presents with often profound behavioral symptomatology. Despite the high burden of these symptoms for both patients and caregivers, there is no general consensus on an effective pharmacological symptomatic treatment. Interestingly, for multiple similar symptoms in primary psychiatric disorders, there is consensus on an effective pharmacological treatment. The aim of this study is to explore currently preferred clinical practices in the pharmacological treatment of specific core behavioral symptoms in bvFTD by world‐leading clinical experts.

**Methods:**

A digital survey was conducted among members of the Neuropsychiatric International Consortium on Frontotemporal Dementia, comprising neurologists, psychiatrists, and neuropsychiatrists. Respondents recommended pharmacological treatments targeting symptoms including disinhibition, apathy, loss of empathy, hyperorality, perseverative/compulsive behaviors, and positive psychotic symptoms.

**Results:**

Of 48 respondents with a median experience of 11.5 years in treating bvFTD, disinhibition was most frequently targeted (58.4%), followed by perseverative/compulsive behaviors (46.5%). Recommended drug classes included atypical antipsychotics (35.1%), selective serotonin reuptake inhibitors (31.2%), antiepileptics (10.0%), serotonin antagonist and reuptake inhibitors (8.4%), benzodiazepines (4.0%), and others (11.4%).

**Conclusions:**

Our survey revealed diverse pharmacological treatment practices for behavioral symptoms in bvFTD, reflecting the expected radical heterogeneity in pharmacological treatment strategies. Notwithstanding this, results from this explorative survey could further inform future research directions, and thus in turn potentially aid in establishing more consensus on effective pharmacological management of bvFTD, while the field awaits the development of highly anticipated disease‐modifying treatment(s).

## INTRODUCTION

Frontotemporal dementia (FTD), caused by frontotemporal lobar degeneration, is the second most common cause of young onset dementia [3, 12, 16]. FTD is characterized by progressive frontal, temporal, and/or insular atrophy [27, 29]. FTD encompasses a heterogeneous spectrum of clinical syndromes, of which behavioral variant FTD (bvFTD) is the most common [18, 23]. bvFTD is characterized by profound and progressive changes in behavior and cognition [6, 21]. In bvFTD, patients often lack any insight into their disease [5, 7, 26].

Presently, there is no curative treatment available for bvFTD [9]. Clearly, the lack of curative treatment poses a serious problem [7]. Concomitantly, on the symptomatic level, there is no consensus on the optimal pharmacological treatment [9]. Currently, clinicians confronted with behavioral symptoms in bvFTD have to treat these pragmatically. As there is no curative treatment for bvFTD expected in the near future, a more effective symptomatic treatment is pivotal to reducing the burden of this disease for both the patients and their caregivers. This burden is reflected by stated unmet needs according to caregivers and patient organizations [7].

Conduction of clinical trials is complicated by the low prevalence of bvFTD, resulting in a few randomized controlled trials (RCTs) of symptomatic treatments. Also, RCTs of symptomatic treatments need to be directed at specific symptoms, as done in clinical practice. Considering the heterogeneity of symptoms in bvFTD, this additionally hampers empirical research and resulting reviews and meta‐analyses. Interestingly, all behavioral symptoms in bvFTD may be similar to behavioral symptoms present in various primary psychiatric disorders (PPDs) [20]. Contrary to bvFTD, in PPD there is a consensus‐based protocol on effective pharmacological treatment for at least some of these behavioral symptoms. Depending on the specific psychiatric disorder, disinhibition can be treated with (atypical) antipsychotics, mood stabilizers, and benzodiazepines [10, 15]. Apathy has, again depending on the specific psychiatric disorder, been proven to be treatable by administering antidepressants [19, 30, 31]. Obsessive–compulsive symptoms can be treated with antidepressants, such as selective serotonin reuptake inhibitors (SSRIs) or clomipramine [1, 4]. Still, in PPD currently no pharmacological treatment is consensus based for executive dysfunction, lack of empathy/sympathy, and hyperorality. Through the comparison to clinical practices in PPDs, in bvFTD more consensus on pharmacological treatment of specific behavioral symptoms may be reached. Importantly, this is exploratory in nature, as comparative studies between behavioral symptoms in bvFTD and possibly similar symptoms in PPDs are lacking. The lack of RCTs and the limitations of current systematic reviews and meta‐analyses substantiate the rationale and methodological approach of this study. Therefore, this study's primary objective is to explore current clinical practice preference in the pharmacological treatment of specific behavioral symptoms in bvFTD using a structured survey. In connection to this, the study's secondary objective is to contribute to consensus on an effective pharmacological treatment. This could enable clinicians to select the most effective pharmacological symptomatic treatment based on collective experience.

## METHODS

### Survey

To assess the current clinically preferred pharmacological treatment for behavioral symptoms in bvFTD, a structured survey was designed using surveymonkey.com, a web‐based survey platform, and consisted of multiple closed and open questions. First, information on the respondent was collected, which included geographical location, professional occupation, and years of experience in the treatment of behavioral symptoms in bvFTD. Second, respondents were asked to recommend three or more pharmacological treatments for one or more of the behavioral symptoms based on their clinical experience. Third, respondents were asked to suggest the most clinically relevant pharmacological treatment to further investigate in a hypothetical future randomized controlled clinical trial. The complete survey can be found as Appendix A to this work.

### Respondents

The survey was distributed among the 96 members of the Neuropsychiatric International Consortium on Frontotemporal Dementia (NIC‐FTD). NIC‐FTD is a growing international collaboration, consisting of world‐leading clinicians in the FTD field, focusing on the neuropsychiatric aspects of frontotemporal dementia. NIC‐FTD consists of neurologists, psychiatrists, neuropsychiatrists, neuropsychologists, and neuroscientists. More information on NIC‐FTD is available via www.nic‐ftd.com.

### Procedure

The 96 NIC‐FTD members received an invitation through email to respond digitally to the survey, which was accessible through the online survey platform SurveyMonkey. Estimated time to fill out the survey was a maximum of 10 min.

### Statistical analysis

Data cleaning and statistical analysis were performed using R, version 4.2.1. (R Core Team [2022]; R: A Language and Environment for Statistical Computing; R Foundation for Statistical Computing, Vienna, Austria; URL: https://www.R‐project.org/). Descriptive data of respondents were presented using frequencies and percentages, and when appropriate, evaluated for normality by assessment of histograms, followed by a Shapiro–Wilk test [22]. Group differences (by occupation) were assessed using analysis of variance models for normally distributed variables, and a Kruskal–Wallis test for nonnormal variables.

### Literature search

Results from the survey were compared to the relevant existing literature. To enable this comparison, a literature search was executed in PubMed. To attain an overview of relevant literature, the search terms were broad and consisted of “frontotemporal dementia” and “treatment.” The search was filtered to generate only systematic reviews. The search yielded 63 articles. The screening of title and/or abstract focused on including only articles on the pharmacological symptomatic treatment of bvFTD. This search specification yielded 14 articles. The full‐text reading focused on including only articles on the pharmacological treatment at the specific behavioral symptom level (i.e., not multiple symptoms or treatment at the syndrome level). This search specification yielded one systematic review, which comprised the literature to which to compare the results of the survey [25].

## RESULTS

### Respondents

**FIGURE 1:**
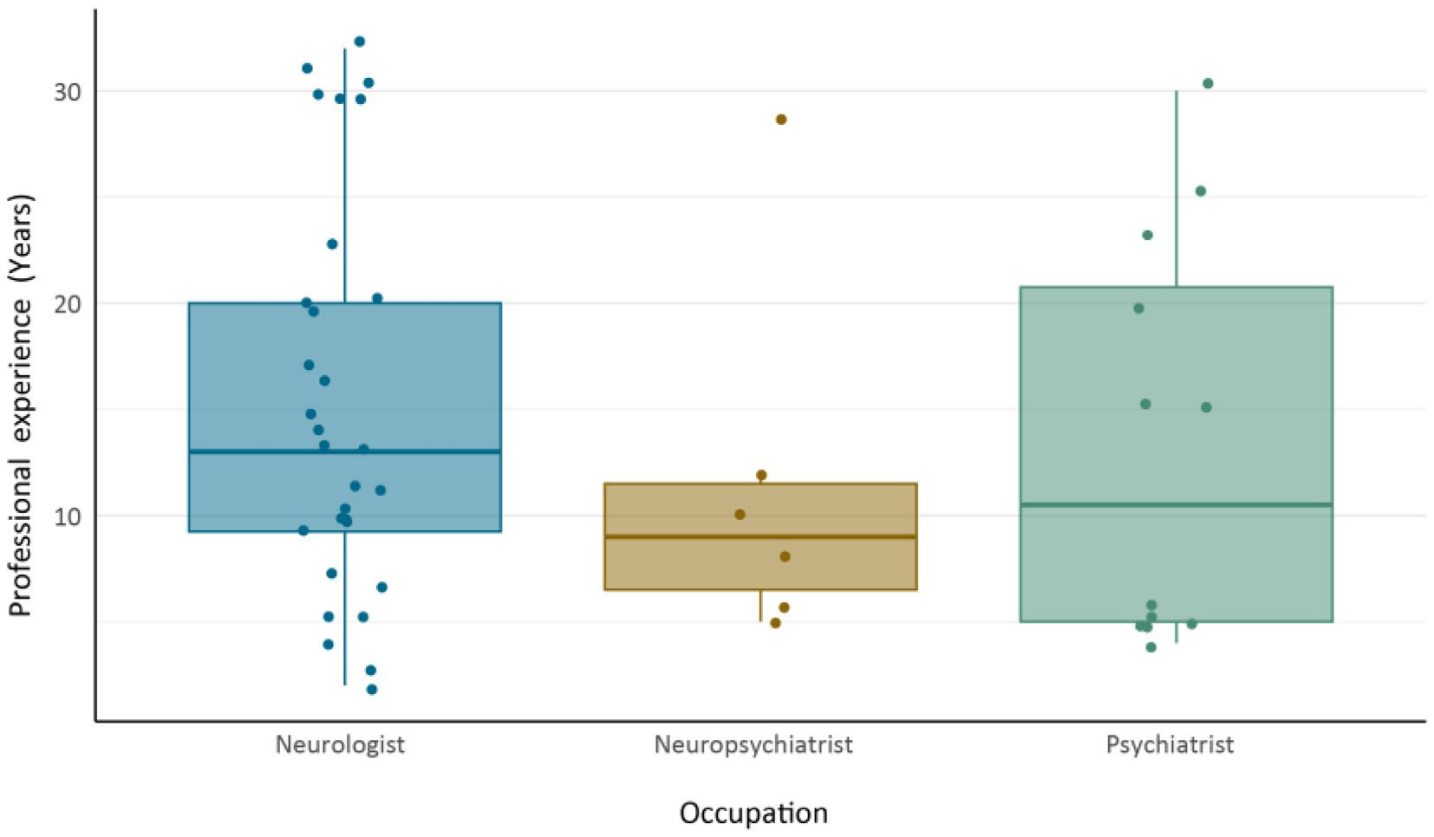
Years of professional experience with pharmacological management of behavioral symptoms in frontotemporal dementia, grouped by occupation.

The complete survey can be found as Appendix A to this work. Responses were collected from NIC‐FTD members between April and September 2023. At the end of data collection, a total of 48 respondents, comprising a response rate of 50.0%, completed the survey: 30 neurologists, 12 psychiatrists, and six neuropsychiatrists (Table 1). Median years of professional experience with pharmacological treatment of behavioral symptoms in FTD was 11.50 years. No significant differences in years of experience were found between occupational groups (Kruskal–Wallis test, *p* = 0.517; Figure 1). To address the possible introduction of bias due to the country of residence of respondents, a separate analysis of responses was performed excluding the biggest group (France). Comparing the results of the total group with this group, proportions of suggested medications differed no more than 5%. Therefore, we present the data of the total group of respondents (*n* = 48) here.

**TABLE 1:** Characteristics of survey respondents.

Respondent characteristic	*n* (%)
Total [overall]	48 (100%)
Occupation	
Neurologist	30 (62.5%)
Neuropsychiatrist	6 (12.5%)
Psychiatrist	12 (25.0%)
Subspecialization	
Behavioral neurology	4 (8.3%)
Cognitive neurology	20 (41.7%)
Geriatric psychiatry	8 (16.7%)
Neuropsychiatry	5 (10.4%)
None/not specified	11 (22.9%)
Country of residence	
Australia	2 (4.2%)
Belgium	1 (2.1%)
Brazil	2 (4.2%)
Canada	4 (8.3%)
Colombia	1 (2.1%)
Finland	2 (4.2%)
France	11 (22.9%)
Germany	2 (4.2%)
India	1 (2.1%)
Italy	7 (14.6%)
Mexico	1 (2.1%)
Netherlands	5 (10.4%)
Portugal	1 (2.1%)
Sweden	1 (2.1%)
Thailand	1 (2.1%)
USA	6 (12.5%)
Professional experience, years, median [IQR]	11.50 [6.00–20.00]

Abbreviation: IQR, interquartile range.

### Current clinical practice tendencies

#### All symptoms

As we allowed each respondent to submit multiple suggestions for pharmacological management of behavioral symptoms, our 48 respondents collectively made 202 suggestions, covering 37 unique medications across 12 drug classes. We grouped suggested medications by drug class and report the five largest groups here: atypical antipsychotics (71/202, 35.1%), SSRIs (63/202, 31.2%), antiepileptics (20/202, 10.0%), serotonin antagonist and reuptake inhibitors (SARIs; 17/202, 8.4%, all trazodone), and benzodiazepines (8/202, 4.0%). Remaining suggested medications were combined under “other” (23/202, 11.4%; Table 2). The most frequently suggested specific medications across all symptoms were quetiapine (29/202, 14.4%), risperidone (22/202, 10.9%), sertraline (21/202, 10.4%), and trazodone (17/202, 8.4%; Table 2).

**TABLE 2:** Suggested prescriptions for pharmacological management of behavioral symptoms in frontotemporal dementia, grouped by specific drug.

Suggested medication	All symptoms, *n* (%)	Behavioral symptoms targeted, *n* (%)					
		Disinhibition	Apathy	Loss of empathy	Compulsive/perseverative behaviors	Hyperorality	Positive psychotic symptoms
All suggestions	**202 (100%)**	**118 (100%)**	**40 (100%)**	**6 (100%)**	**94 (100%)**	**32 (100%)**	**69 (100%)**
Atypical antipsychotics	**71 (35.1%)**	**55 (46.6%)**	**0 (0.0%)**	**0 (0.0%)**	**24 (25.5%)**	**6 (18.8%)**	**60 (87.0%)**
Aripiprazole	4 (2.0%)	3 (2.5%)	‐	‐	‐	‐	4 (5.8%)
Brexpiprazole	1 (0.5%)	1 (0.8%)	‐	‐	‐	‐	1 (1.4%)
Olanzapine	15 (7.4%)	12 (10.2%)	‐	‐	5 (5.3%)	‐	13 (18.8%)
Quetiapine	29 (14.4%)	24 (20.3%)	‐	‐	10 (10.6%)	3 (9.4%)	22 (31.9%)
Risperidone	22 (10.9%)	15 (12.7%)	‐	‐	9 (9.6%)	3 (9.4%)	20 (29.0%)
SSRIs	**63 (31.2%)**	**26 (22.0%)**	**29 (72.5%)**	**3 (50.0%)**	**44 (46.8%)**	**15 (46.9%)**	**0 (0.0%)**
Citalopram	13 (6.4%)	5 (4.2%)	5 (12.5%)	1 (16.7%)	10 (10.6%)	2 (6.3%)	‐
Escitalopram	14 (6.9%)	4 (3.4%)	9 (22.5%)	1 (16.7%)	10 (10.6%)	4 (12.5%)	‐
Fluoxetine	3 (1.5%)	2 (1.7%)	‐	‐	3 (3.2%)	2 (6.3%)	‐
Fluvoxamine	3 (1.5%)	1 (0.8%)	2 (5.0%)	‐	2 (2.1%)	1 (3.1%)	‐
Paroxetine	8 (4.0%)	5 (4.2%)	2 (5.0%)	‐	5 (5.3%)	2 (6.3%)	‐
Sertraline	21 (10.4%)	9 (7.6%)	10 (25%)	1 (16.7%)	14 (14.9%)	4 (12.5%)	‐
Vortioxetine	1 (0.5%)	‐	1 (2.5%)	‐	‐	‐	‐
Antiepileptics	**20 (9.9%)**	**17 (14.4%)**	**0 (0.0%)**	**0 (0.0%)**	**6 (6.4%)**	**2 (6.3%)**	**4 (5.8%)**
Gabapentin	3 (1.5%)	3 (2.5%)	‐	‐	1 (1.1%)	‐	‐
Lamotrigine	2 (1.0%)	2 (1.7%)	‐	‐	1 (1.1%)	‐	1 (1.4%)
Levetiracetam	2 (1.0)	1 (0.8%)	‐	‐	‐	‐	‐
Topiramate	1 (0.5%)	‐	‐	‐	‐	1 (3.1%)	‐
Valproate	12 (5.9%)	11 (9.3%)	‐	‐	4 (4.3%)	1 (3.1%)	3 (4.3%)
SARIs	**17 (8.4%)**	**13 (11.0%)**	**0 (0.0%)**	**0 (0.0%)**	**13 (13.8%)**	**9 (28.1%)**	**0 (0.0%)**
Trazodone	17 (8.4%)	13 (11.0%)	0 (0.0%)	0 (0.0%)	13 (13.8%)	9 (28.1%)	0 (0.0%)
Benzodiazepines	**8 (4.0%)**	**4 (3.4%)**	**0 (0.0%)**	**0 (0.0%)**	**4 (4.3%)**	**0 (0.0%)**	**1 (1.4%)**
Clonazepam	1 (0.5%)	‐	‐	‐	1 (1.1%)	‐	‐
Lorazepam	4 (2.0%)	2 (1.7%)	‐	‐	2 (2.1%)	‐	‐
Oxazepam	3 (1.5%)	2 (1.7%)	‐	‐	1 (1.1%)	‐	1 (1.4%)
Other	**23 (11.4%)**	**3 (2.5%)**	**11 (27.5%)**	**3 (50.0%)**	**3 (3.2%)**	**0 (0.0%)**	**4 (5.8%)**
Amantadine	1 (0.5%)	‐	1 (2.5%)	‐	‐	‐	‐
Bupropion	1 (0.5%)	‐	1 (2.5%)	‐	‐	‐	‐
Donepezil	1 (0.5%)	‐	‐	‐	‐	‐	‐
Etifoxine	1 (0.5%)	‐	‐	‐	1 (1.1%)	‐	‐
Haloperidol	1 (0.5%)	‐	‐	‐	‐	‐	1 (1.4%)
Lemborexant	1 (0.5%)	‐	‐	‐	‐	‐	‐
Lithium	1 (0.5%)	1 (0.8%)	‐	‐	‐	‐	‐
Memantine	1 (0.5%)	1 (0.8%)	‐	‐	1 (1.1%)	‐	‐
Mirtazapine	2 (1.0%)	‐	‐	‐	‐	‐	‐
Modafinil	1 (0.5%)	‐	1 (2.5%)	‐	‐	‐	‐
Oxytocin	1 (0.5%)	‐	1 (2.5%)	1 (16.7%)	‐	‐	‐
Promazine	2 (1.0%)	1 (0.8%)	‐	‐	‐	‐	2 (2.9%)
Rivastigmine	2 (1.0%)	‐	‐	‐	‐	‐	1 (1.4%)
Rotigotine	1 (0.5%)	‐	1 (2.5%)	‐	‐	‐	‐
Venlafaxine	6 (3.0%)	‐	6 (15.0%)	2 (33.3%)	1 (1.1%)	‐	‐

Abbreviations: SARI, serotonin antagonist and reuptake inhibitor; SSRI, selective serotonin reuptake inhibitor.

#### Results per targeted behavioral symptom

Each suggested treatment option targeted one or more behavioral symptoms. A total of 118 of 202 suggestions (58.4%), made collectively by our 48 respondents, most frequently mentioned disinhibition as a therapeutic target. Disinhibition was most frequently suggested to be treated with atypical antipsychotics (55/118, 46.6%). Specific medications suggested to treat disinhibition were quetiapine (24/118, 20.3%), risperidone (15/118, 12.7%), and trazodone (13/118, 11.0%). Compulsive or perseverative behaviors were targeted by 94 of 202 suggestions (46.5%), most frequently by SSRIs (44/94, 72.5%). Specific medications suggested to treat this symptom were sertraline (14/94, 14.9%), trazodone (13/94, 13.8%), and quetiapine, citalopram, or escitalopram (all 10/94, 10.6%). Positive psychotic symptoms (PPSs) were targeted by 69 of 202 suggested medications (34.2%), most frequently by atypical antipsychotics (60/69, 87.0%). Specific medications suggested to treat PPSs were quetiapine (22/69, 31.9%), risperidone (20/69, 29.0%), and olanzapine (13/69, 18.8%). Apathy was targeted by 40 of 202 suggestions (19.8%), most frequently by SSRIs (29/40, 72.5%). Specific medications suggested to treat apathy were sertraline (10/40, 25.0%), escitalopram (9/40, 22.5%), and venlafaxine (6/40, 15.0%). Hyperorality was targeted by 32 of 202 (15.8%) suggested medications, most frequently by SSRIs (15/32, 46.9%). Specific medications suggested to treat hyperorality were trazodone (9/32, 28.1%), sertraline (4/32, 12.5%), and escitalopram (4/32, 12.5%). Finally, loss of empathy was targeted by only six of 202 suggestions (3.0%), most frequently by SSRIs (3/6, 50.0%). Specific medications targeting loss of empathy were venlafaxine (2/6, 33.3%) and citalopram, escitalopram, sertraline, or oxytocin (all 1/6, 16.7%). These results, as well as less frequently suggested medications, are depicted in Table 2.

#### Results per drug class

**FIGURE 2:**
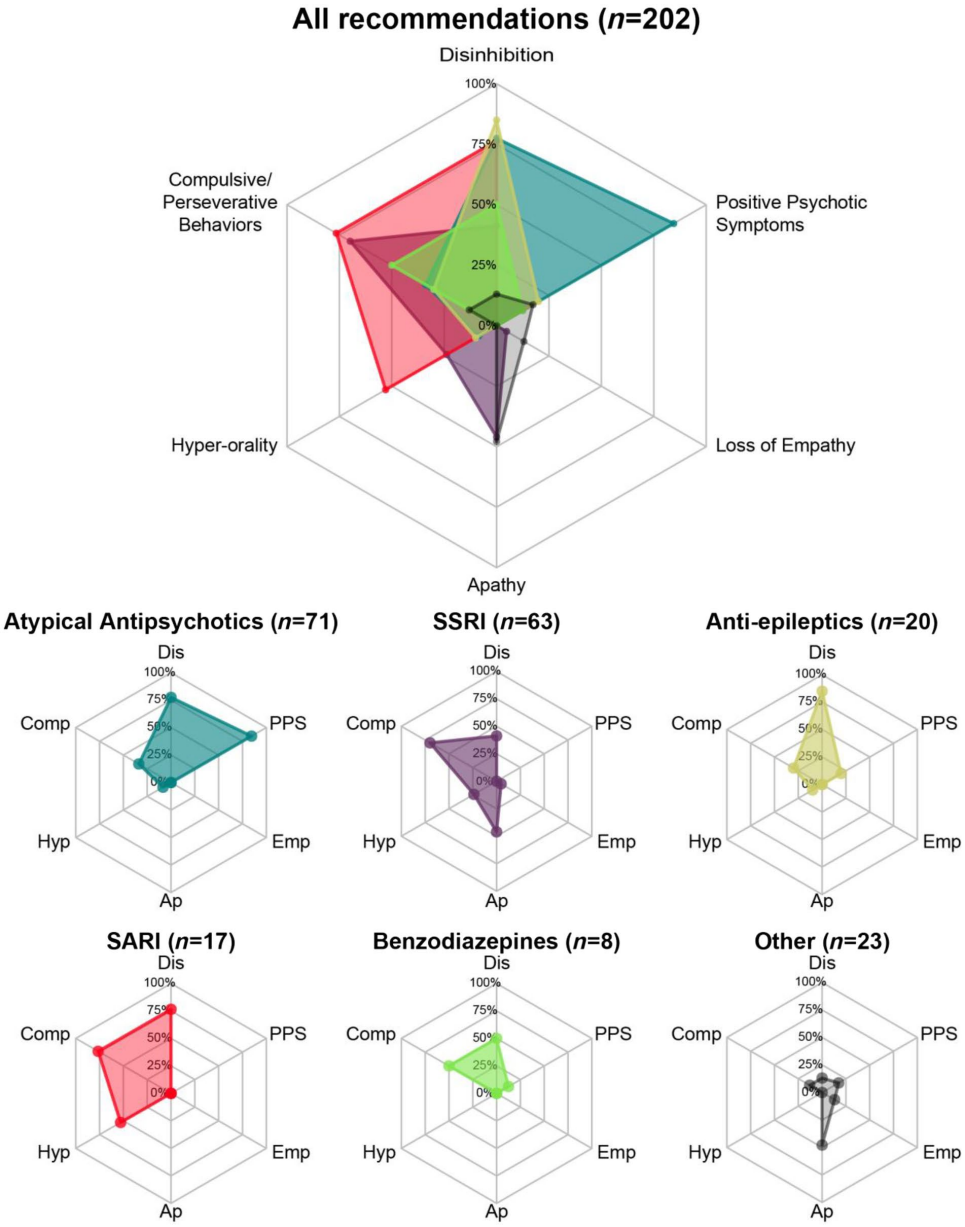
Radar plots of suggested prescriptions for pharmacological management of behavioral symptoms in frontotemporal dementia. Ap, apathy; Comp, compulsive/perseverative behaviors; Dis, disinhibition; Emp, loss of empathy; Hyp, hyperorality; PPS, positive psychotic symptoms (hallucinations/delusions); SARI, serotonin antagonist and reuptake inhibitors; SSRI, selective serotonin reuptake inhibitors.

Suggested treatment options revealed clinical practice tendencies per drug class, targeting behavioral symptoms. Figure 2 depicts radar plots of these preferred indications. Of 71 suggestions with atypical antipsychotics, most targeted disinhibition and PPSs (55/71, 77.5% and 60/71, 84.5%, respectively).

Of 63 suggestions with SSRIs, most targeted compulsive/perseverative behaviors and apathy (44/63, 69.8% and 29/63, 41.2%, respectively). Of 20 suggestions with antiepileptics, mainly disinhibition was targeted (17/20, 85.0%). Of 17 suggestions with SARIs (all trazodone), most targeted disinhibition and compulsive/perseverative behaviors (both 13/17, 76.5%). Of eight suggestions with benzodiazepines, most targeted symptoms were disinhibition and compulsive/perseverative behaviors (both 4/8, 50.0%).

#### Hypothetical future trial

The following results are in response to our survey's last question, regarding a hypothetical future study assessing which drug–symptom combination would be relevant to investigate. Disinhibition was most frequently suggested to be the symptomatic target (18/48, 37.5%), followed by apathy (15/48, 31.3%). The most frequently suggested drug class was SSRIs (12/48, 25%), followed by atypical antipsychotics (8/48, 16.7%). The most mentioned symptom–treatment combination was SSRIs aiming to treat disinhibition (7/48, 14.6%; Table 3).

**TABLE 3:** Results of respondents' suggestions for a hypothetical future randomized controlled trial managing behavioral symptoms in behavioral variant frontotemporal dementia using currently licensed medications.

Drug class	Behavioral symptom targeted, *n* (%)						Total, *n* (%)
	Disinhibition	Apathy	Loss of empathy	Compulsive/perseverative behaviors	Hyperorality	Positive psychotic symptoms	
Antidepressants							
SSRIs	7 (14.6%)	‐	1 (2.1%)	3 (6.3%)	1 (2.1%)	‐	**12 (25.0%)**
SNRIs	1 (2.1%)	1 (2.1%)	‐	‐	‐	‐	**2 (4.2%)**
Other	4 (8.3%)	2 (4.2%)	‐	‐	‐	‐	**6 (12.5%)**
Antipsychotics							
Typical	‐	‐	‐	1 (2.1%)	‐	‐	**1 (2.1%)**
Atypical	5 (10.4%)	‐	‐	‐	‐	3 (6.3%)	**8 (16.7%)**
Antiepileptics	‐	‐	‐	1 (2.1%)	2 (4.2%)	‐	**3 (6.3%)**
Dopamine agonists	‐	2 (4.2%)	‐	‐	‐	‐	**2 (4.2%)**
NMDA antagonists	‐	1 (2.1%)	‐	‐	‐	‐	**1 (2.1%)**
Psychostimulants	‐	3 (6.3%)	‐	‐	‐	‐	**3 (6.3%)**
Other	1 (2.1%)	6 (12.5%)	1 (2.1%)	1 (2.1%)	‐	1 (2.1%)	**10 (20.8%)**
Total	**18 (37.5%)**	**15 (31.3%)**	**2 (4.2%)**	**6 (12.5%)**	**3 (6.3%)**	**4 (8.3%)**	**48 (100%)**

Abbreviations: NMDA, N‐methyl‐D‐aspartate; SSRI, selective serotonin reuptake inhibitor; SNRI, serotonin and norepinephrine reuptake inhibitor.

## DISCUSSION

This study's primary objective was to assess the current clinically preferred pharmacological treatment of specific behavioral symptoms in the context of bvFTD. In recapitulation, the most frequently treated specific behavioral symptom was disinhibition, followed by compulsive/perseverative behaviors and PPSs. Other behavioral symptoms, such as apathy, hyperorality, and loss of empathy, were less frequently pharmacologically treated. Caregivers report loss of emotional connection, preoccupation and restlessness, and apathy as the major relevant themes causative of increased burden [7]. Notably, this burden is only partly reflected in the results here. This mostly seems to be reflecting lack of effective pharmacological treatment for both loss of empathy and apathy.

Notwithstanding expected heterogeneity, in the suggestions from our survey, multiple patterns can be detected. First, these patterns in suggestions on a symptom level are here compared to the relevant systematic review on pharmacological treatment of specific behavioral symptoms in bvFTD. As literature on pharmacological treatment of behavioral symptoms in bvFTD is scarce, especially on a specific symptom level, contextualizing the results may be complicated. Interpretation of this context should thus be cautious. Second, on a specific symptom level, potential congruency between these suggestions and treatment of possibly similar symptoms in clinical psychiatric practice is explored.

First, disinhibition was mostly targeted with atypical antipsychotics, such as quetiapine. This clear result is remarkable, as it is not recommended in the relevant literature [25]. In comparison to a manic episode in, for example, bipolar disorder, this is, however, mostly congruent to clinical practice in psychiatry [28]. Second, although suggested medications targeting compulsive/perseverative behaviors were somewhat heterogeneous, SSRIs are clearly most selected, as these comprise about half of the recommendations. This result largely aligns with the relevant literature, which suggests an effect of the antidepressants SSRIs and tricyclic antidepressants [25]. In comparison to potentially similar symptoms in obsessive–compulsive disorder (OCD), this is largely congruent with clinical practice in psychiatry, as OCD is always primarily treated with SSRIs, aside from nonpharmacological treatments [1]. Third, PPSs (i.e., hallucinations and delusions) are treated homogeneously, with atypical antipsychotics selected in almost all of respondents' suggestions. Although clinically burdensome, PPSs are not discussed in the literature, which hampers comparison [25]. Still, this only strengthens the clinical relevance of this result. In comparison to a potentially similar psychotic decompensation in a schizophrenic disorder, this is congruent with clinical practice in psychiatry [11]. Fourth, suggestions targeting apathy consisted primarily of SSRIs and serotonin and norepinephrine reuptake inhibitors (SNRIs). This is not congruent with the literature, which again is heterogeneous but clearly does not suggest SSRIs and SNRIs, as instead, the few RCTs done have shown effect of agomelatine, oxytocin, and stimulants [25]. Notably, in comparison to the possibly similar apathy in major depressive disorder, this is congruent with clinical practice in psychiatry, where SSRIs and SNRIs are the first step in its pharmacological treatment [2]. It is known that apathy is challenging to treat in PPDs as well [19, 30, 31]. This is congruent with the result here that less pharmacological suggestions were directed at apathy. Fifth, hyperorality was also most frequently suggested to be treated with SSRIs. This result is congruent with the literature, with SSRIs as its primary suggestion [25]. In comparison to the potentially similar compulsive binge eating in bulimia nervosa, this is congruent with clinical practice in psychiatry, where this symptom can be treated with SSRIs [13]. Sixth, treating loss of empathy pharmacologically was almost never mentioned. This result is congruent with the literature, which also rarely suggests a pharmacological treatment for loss of empathy [25]. This is in line with clinical practice in psychiatry, in which there is also no known effective pharmacological treatment for a possibly similar symptom [17, 24]. Small studies on oxytocin have already shown some promising results in the treatment of both apathy and lack of empathy [8, 14] but remain to be proven in larger clinical cohorts.

In a deduction of patterns that go beyond the behavioral symptom level, the most frequently suggested specific pharmacological treatment was quetiapine, which was selected for multiple behavioral symptoms, mainly disinhibition and PPSs. This is in line with clinical practice in psychiatry, as its primary indications are PPSs (such as in psychotic disorders) and manic episodes (such as in bipolar disorder) [28]. Notably, in the SARI group, trazodone was suggested to target three different behavioral symptoms simultaneously: disinhibition, compulsive/perseverative behavior, and hyperorality. This specific multitargeted prescription was unique to trazodone compared with other suggested pharmacological treatments. Still, this result should be interpreted with some reservation, as trazodone was suggested relatively infrequently (17/202 suggestions), and multiple other pharmacological treatment classes were prescribed more often for specific behavioral symptoms, such as SSRIs for compulsive/perseverative behaviors and atypical antipsychotics or antiepileptics for disinhibition. Importantly, the unique recommendation of trazodone to treat multiple behavioral symptoms is substantiated in the literature, as it is stated that altered serotonergic systems in bvFTD may contribute to loss of empathy, disinhibition, and apathy simultaneously, thus possibly potentiating SSRIs/SARIs to treat multiple behavioral symptoms simultaneously [9, 25].

For future directions of research, with a focus on randomized controlled clinical trial design, recommendations varied widely, but the most suggested pharmacological treatment to investigate was an SSRI to treat disinhibition. Also, as was evident from the results, and in line with both the existing literature and psychiatric practice, apathy is challenging to treat and was put forward by respondents relatively often to investigate in a future clinical trial (31.3% of respondents) [19, 30, 31]. Next, although lack of empathy is often stated to be an important contributor to caregiver burden, almost none of the respondents suggested this as a focus of future clinical trials [7]. This may be somewhat unexpected, as multiple small studies on oxytocin seemed relatively promising [8, 14].

In this study, there are multiple limitations to consider. First, this survey only focused on preferred pharmacological treatment and did not include nonpharmacological treatment in clinical and first‐line care settings. Second, although the comparison of the results to possibly similar symptoms in psychiatric practice may be relevant, comparative studies are practically nonexistent. Thus, this comparison should always be interpreted with utmost caution. In the survey, dosage was not considered as a factor. This is important to note, because it its known that the same pharmacological treatment can be dosed higher to treat PPDs compared with neurodegenerative disease. Thus, the analogy with psychiatric clinical practice should be considered with some reservation. Third, there was a disproportionate representation of respondents from certain countries, which is an anticipated limitation when conducting a survey, as it may have influenced the results. However, when comparing our results to a subset with the largest group (French respondents) excluded, results were similar to those from the total group of respondents. Fourth, results of this study follow from a digital survey, based on pragmatic experiences of clinicians. Thus, even if consensus is reached due to this collective experience, it is still pragmatic knowledge. Fifth, there was heterogeneity in the responses to consider in the analysis of the data. The structure of the digital survey, which facilitated multiple responses per item, led to a heterogeneity per respondent. Ideally, there is homogeneity between the respondents, as their experience is thus represented in an equal manner. Still, this structure was considered beforehand to optimize input. Also, this structure fits the explorative character of this study well.

There are multiple strengths to mention. First, the study focused on a clinically relevant gap in knowledge through assessment of combined expert opinion. Second, the method of a survey allowed a direct assessment of the clinician's experience and thus representative data. In connection to this, third, digital distribution of the survey proved to be an accessible and concise way to collect responses in a low‐friction manner, which in turn explains the high number of respondents. Fourth, the respondents varied in medical specializations, thus constituting a representative population.

In conclusion, this study presented the preferred clinical practice tendencies of pharmacological treatment of specific behavioral symptoms in bvFTD, which reflected the expected heterogeneity described in previous literature. Notwithstanding this, results from this explorative survey among the world's leading academic centers can inform the direction of future studies as well as aid in establishing consensus on effective pharmacological symptomatic treatment, as the field awaits the arrival of anticipated disease‐modifying treatment(s).

## AUTHOR CONTRIBUTIONS

**Dirk van Paassen:** Conceptualization; methodology; investigation; writing – original draft; writing – review and editing; project administration. **Luc Hartog:** Formal analysis; data curation; conceptualization; methodology; writing – review and editing; visualization; project administration; writing – original draft. **Sterre de Boer:** Writing – review and editing. **Everard Vijverberg:** Writing – review and editing. **Simon Ducharme:** Writing – review and editing; resources. **Yolande Pijnenburg:** Writing – review and editing; supervision; resources. **Alexander Santillo:** Writing – review and editing; supervision; conceptualization; methodology.

## CONFLICT OF INTEREST STATEMENT

None of the authors has any conflict of interest to disclose.

## Data Availability

“Anonymized data will be shared by request from a qualified academic investigator for the sole purpose of replicating procedures and results presented in the article if data transfer is in agreement with relevant legislation on the general data protection regulation and decisions and by the relevant Ethical Review Boards, which should be regulated in a material transfer agreement.”.

## APPENDIX A

### PREFERRED CLINICAL PRACTICE SURVEY

*Page 1: Respondent information*

- Email: ______________________
- Affiliation:______________________
- Occupation:______________________
- Subspecialization:______________________
- Experience with pharmacological management of behavioral symptoms in bvFTD?Yes / No
- Years of professional experience?______________________

*Page 2: Recommendations/suggestions for pharmacological management of behavioral symptoms in bvFTD*

On this page, we ask you to select three preferred drug classes for treating behavioral symptoms in bvFTD (not hierarchical). Furthermore, we are interested in your experience with specific drug choices in treating these symptoms.

- Drug class 1 (select one):
  - SSRIs, SNRIs, typical antipsychotics, atypical antipsychotics, antiepileptics, benzodiazepines, cholinesterase inhibitors, dopamine agonists, NMDA antagonists, Oxytocin agonists, other (please specify).
  _________________
- With what specific drug from this class do you have experience as a treatment option for behavioral symptoms in bvFTD? _________________
- What symptom (or symptoms) do you intend to treat by prescribing this drug?
  - Disinhibition, apathy/inertia, loss of empathy/sympathy, compulsive/perseverative behaviors, hyperorality, hallucinations/delusions
- Drug class 2 (select one):
- SSRIs, SNRIs, typical antipsychotics, atypical antipsychotics, antiepileptics, benzodiazepines, cholinesterase inhibitors, dopamine agonists, NMDA antagonists, oxytocin agonists, other (please specify). _________________
- With what specific drug from this class do you have experience as a treatment option for behavioral symptoms in bvFTD? _________________
- What symptom (or symptoms) do you intend to treat by prescribing this drug?
  - Disinhibition, apathy/inertia, loss of empathy/sympathy, compulsive/perseverative behaviors, hyperorality, hallucinations/delusions
- Drug class 3 (select one):
  - SSRIs, SNRIs, typical antipsychotics, atypical antipsychotics, antiepileptics, benzodiazepines, cholinesterase inhibitors, dopamine agonists, NMDA antagonists, oxytocin agonists, other (please specify).
  _________________
- With what specific drug from this class do you have experience as a treatment option for behavioral symptoms in bvFTD? _________________
- What symptom (or symptoms) do you intend to treat by prescribing this drug?
  - Disinhibition, apathy/inertia, loss of empathy/sympathy, compulsive/perseverative behaviors, hyperorality, hallucinations/delusions

*Page 3: Future RCT investigating pharmacological treatment options for behavioral symptoms in bvFTD*

If you were to design an RCT targeted at behavioral symptoms in bvFTD using currently licensed drugs, which symptom would you treat and what drug would you use?

- Symptom to be treated (select one): disinhibition, apathy/inertia, loss of empathy/sympathy, compulsive/perseverative behaviors, hyperorality, hallucinations/delusions
- Drug of interest: _________________

Abbreviations: bvFTD, behavioral variant frontotemporal dementia; NMDA, N‐methyl‐D‐aspartate; RCT, randomized controlled trial; SNRI, serotonin and norepinephrine reuptake inhibitor; SSRI, selective serotonin reuptake inhibitor.
